# Influence of Diclofenac on Activated Sludge Bacterial Communities in Fed-Batch Reactors

**DOI:** 10.17113/ftb.58.04.20.6424

**Published:** 2020-12

**Authors:** Barbara Kraigher, Ines Mandic-Mulec

**Affiliations:** University of Ljubljana, Biotechnical Faculty, Department of Food Science and Technology, Chair of Microbiology, Večna pot 111, 1000 Ljubljana, Slovenia

**Keywords:** activated sludge, pharmaceuticals, diclofenac, bacterial community T-RFLP, *Pseudomonas*

## Abstract

**Research background:**

The occurrence and environmental toxicity of pharmaceuticals have recently attracted increasing attention. Diclofenac is a highly consumed non-steroidal anti-inflammatory drug, which is often detected in wastewaters, but investigations of its influence on bacteria are scarce.

**Experimental approach:**

We investigated the influence of this pharmaceutical on bacterial community in activated sludge exposed to increasing concentrations of diclofenac in fed-batch reactors over 41 days. Nitrification activity of the activated sludge was measured and changes in bacterial community structure were followed using culture-independent molecular method (terminal restriction fragment length polymorphism, T-RFLP) and by the cultivation approach.

**Results and conclusions:**

Nitrification activity was not detectably influenced by the addition of diclofenac, while the main change of the bacterial community structure was detected only at the end of incubation (after 41 days) when diclofenac was added to artificial wastewater as the only carbon source. Changes in community composition due to enrichment were observed using cultivation approach. However, taxonomic affiliation of isolates did not match taxons identified by T-RFLP community profiling. Isolates obtained from activated sludge used as inoculum belonged to five genera: *Comamonas*, *Arthrobacter*, *Acinetobacte*r, *Citrobacter* and *Aeromonas*, known for their potential to degrade aromatic compounds. However, only *Pseudomonas* species were isolated after the last enrichment step on minimal agar plates with diclofenac added as the sole carbon source.

**Novelty and scientific contribution:**

Our results suggest that the selected recalcitrant and commonly detected pharmaceutical does not strongly influence the sensitive and important nitrification process of wastewater treatment. Moreover, the isolated strains obtained after enrichment procedure that were able to grow on minimal agar plates with diclofenac added as the only carbon source could serve as potential model bacteria to study bacterial diclofenac degradation.

## INTRODUCTION

Pharmaceuticals, generally regarded as being extremely advantageous for increasing human life expectancy, on the other hand represent a serious burden for the environment. Worldwide consumption rates of hundreds of tonnes of pharmaceuticals have a potential to cause undesirable ecological and human health effects (*1*-*3*). The pharmaceutical industry has increased enormously in recent years and currently represents one of the most profitable branches of industry. Consequently, substantial amounts of pharmaceuticals can reach and impact the environment, either through direct discharge of pharmaceuticals or due to inefficient elimination in wastewater treatment plants (WWTPs). Several studies have demonstrated that some pharmaceuticals are efficiently eliminated by WWTPs (ibuprofen, naproxen, ketoprofen), while others are resistant to biodegradation (diclofenac, clofibric acid) (*4*-*7*). Although many pharmaceuticals and their metabolites have been detected in aquatic environments and even in drinking water samples at concentrations ranging from ng/L up to hundreds of μg/L (*4*, *8*-*10*), there still remains a limited understanding of the extent and magnitude of their ecological effects.

Diclofenac is a non-steroidal anti-inflammatory drug (NSAID) prescribed in daily doses of 75–150 mg to reduce inflammation. It acts as an analgesic to reduce pain in patients with arthritis or acute injury and can even be used to reduce menstrual pain (*11*). Over the counter use of diclofenac is approved in some countries for minor pains and fever associated with common infections and it has been estimated that 940 tonnes are consumed annually worldwide (*11*). Chemically, diclofenac is 2-[(2,6-dichlorophenyl)amino]-benzeneacetic acid. One benzene ring is chlorinated, which contributes to the recalcitrant nature of this pharmaceutical for biological degradation. The highly variable and relatively low biodegradation of diclofenac in WWTPs has been reported, mainly in the range of 21-40% (*11*). In the aquatic environment, diclofenac is one of the most frequently detected pharmaceuticals (*12*). It has been detected in drinking water (*12*) and has caused the death of millions of vultures in Southeast Asia through its use in veterinary medicine (*13*, *14*). In the liver, kidneys and gills of rainbow trout, cytopathology occurred already at concentrations of 1 μg/L (*15*). Studies of diclofenac influence on microbial communities are scarce. Microorganisms in lotic biofilms were inhibited at diclofenac concentrations of 100 μg/L (*16*). At very high concentrations (50-100 mg/L), it was shown to inhibit bacterial growth in most of the tested Gram-positive and Gram-negative strains by inhibiting DNA synthesis (*17*).

In our previous studies (*18*, *19*), we have shown that a mixture of selected commonly used pharmaceuticals including diclofenac caused shifts in the community structure of activated sludge in pilot bioreactors. The aim of this study is to evaluate the effect of diclofenac, the most recalcitrant drug in the pharmaceutical mixture (*5*), on the nitrification activity and the community structure of activated sludge in fed-batch reactors exposed to increasing concentration of diclofenac. Additionally, we used the enrichment culture approach to isolate bacteria that are capable of growing on minimal agar plates with diclofenac added as the only carbon source.

## MATERIALS AND METHODS

### Experimental setup and sampling

Batch cultures of activated sludge were prepared in 500-mL Erlenmeyer flasks containing 200 mL of artificial wastewater, which was prepared by dissolving a mineral nutrient composition in tap water, simulating the composition of real municipal wastewater (*5*). The following chemicals were used: yeast extract (130 mg/L), casein peptone (130 mg/L), meat extract (130 mg/L; all Biolife Italiana, Milano, Italy), CH_3_COONH_4_ (317 mg/L), NH_4_Cl (40 mg/L; both Kemika, Zagreb, Croatia), K_2_HPO_4_ (24 mg/L), KH_2_PO_4_ (8 mg/L), CaCO_3_ (100 mg/L), MgCO_3_ (100 mg/L), NaCl (40 mg/L; all Sigma-Aldrich, Merck, Gillingham, UK), and FeSO_4_·7H_2_O (5 mg/L; Merck, Darmstadt, Germany). Each flask (fed-batch reactor) was inoculated with 20 mL of activated sludge obtained from the R200 reactor described by Kraigher *et al.* (*18*), which was operated for 18 months with artificial wastewater containing 200 μg/L of each of the following pharmaceuticals: diclofenac, ibuprofen, naproxen, ketoprofen and clofibric acid. This sludge was selected because it contained a biomass already adapted to the selected concentration of pharmaceuticals and it was expected that potential changes in community structure and enrichment of bacteria capable of degrading diclofenac would be detected sooner. Fed-batch reactors were incubated at 220 rpm and 28 °C in the darkness. Activated sludge was exposed to either 200 μg/L diclofenac (Sigma-Aldrich, Merck) throughout the entire experiment, or to 5 mg/L diclofenac (with increasing concentrations from 1 to 5 mg/L in the first 10 days of incubation). One fed-batch reactor was incubated without the addition of pharmaceuticals. The fed-batch reactors were operated in the following way throughout the experiment: the hydraulic retention time of two days was maintained by settling down the sludge every two days (three days over the weekends), decanting the medium, and adding fresh artificial wastewater with (or without) diclofenac. Each time the samples of influents (artificial wastewater) and effluents (decanted medium) were taken and kept frozen until N-NH_4_^+^ and N-(NO_2_^-^+NO_3_^-^) concentrations were analysed by continuous flow analyser (FlowSys Alliance Instruments, Salzburg, Austria). After 20 days of incubation, the artificial wastewater without organic carbon (*i.e.* without casein peptone, CH_3_COONH_4_, meat and yeast extract) was used to feed the fed-batch reactors which were incubated for additional three weeks. Activated sludge for bacterial community analyses was sampled (1 mL) at indicated time intervals and stored at –80 °C until DNA extraction.

### Bacterial community analyses

Terminal restriction fragment length polymorphism (T-RFLP) analysis was used to compare bacterial community structures. DNA was extracted from 1-mL subsamples (two independent DNA isolations were obtained for each sample) using the UltraClean soil DNA isolation kit (MoBio Solano Beach, CA, USA) according to the manufacturer’s instructions. Isolated total community DNA (concentration of 50-100 ng/μL) was checked on a 1% agarose gel and compared to the Gene Ruler DNA Ladder Mix (Thermo Fisher Scientific Baltics, UAB, Vilnius, Lithuania) to estimate the size and concentration. Purified DNA was then used as a template for polymerase chain reaction (PCR) with 16S rRNA gene primers 27f labelled with 6-FAM (6-carboxyfluorescein) at the 5’ end and 927r (*20*) for the total community analyses, following the protocols described by Kraigher *et al.* (*18*). Restriction with restriction enzyme *Msp*I (Thermo Fisher Scientific Baltics, UAB), ethanol precipitation and T-RFLP profiling were performed. Profiles were generated using ABI PRISM 310 DNA sequencer and GeneScan analysis software (*21*). Terminal restriction fragments (T-RFs) with peak heights of less than 50 fluorescence units and T-RFs that were less than 50 bp long were excluded from the analyses. T-RFLP profiles of duplicate DNA isolations per sample were analysed using the BioNumerics program v. 6.6 (*22*). Normalized intensity values and the positions of the detected bands were used for cluster analysis, while peaks representing less than 1% of the total fluorescence of all peaks in a sample were excluded from the analysis. Pearson’s correlation coefficient, which considers both fragment length and peak height, was used to calculate similarity coefficients. A dendrogram was then constructed using the unweighted pair-group method with arithmetic means (UPGMA) algorithm.

### Isolation of bacteria capable of growing on minimal plates with diclofenac

Bacteria were isolated from the activated sludge that was used as inoculum for the incubation experiment and from the fed-batch cultures at the end of the experiment (after 41 days of incubation with diclofenac, with the last 20 days without the addition of any other external carbon source except for diclofenac). Mineral medium (MM) agar plates supplied with 20 mg/L of diclofenac as the only carbon and energy sources were used for isolation. MM agar plates consisted of (in g/L): 3.5 Na_2_HPO_4_·2H_2_O (Sigma-Aldrich, Merck), 1 KH_2_PO_4_ (Sigma-Aldrich, Merck), 0.5 (NH_4_)_2_SO_4_·2H_2_O (Merck), 0.1 MgCl_2_·6H_2_O (Honeywell-Fluka, Basel, Switzerland), 0.05 Ca(NO_3_)_2_·4H_2_O (Honeywell-Fluka), 15 highly purified agar (Biolife Italiana) at pH=7.25 and finally 1 mL trace element solution SL-4 was added. The trace element solution SL-4 consisted of (in g/L): 0.5 EDTA (Sigma-Aldrich, Merck), 0.2 FeSO_4_·7H_2_O (Merck), 0.01 ZnSO_4_·7H_2_O (Merck), 0.003 MnCl_2_·4H_2_O (Merck), 0.03 H_3_BO_3_ (Merck), 0.02 g CoCl_2_·6H_2_O (Honeywell-Fluka), 0.001 g CuCl_2_·2H_2_O (Sigma-Aldrich, Merck), 0.002 NiCl_2_·6H_2_O (Sigma-Aldrich, Merck) and 0.003 Na_2_MoO_4_·2H_2_O (Kemika). The activated sludge was centrifuged, washed with MM medium and then different dilutions were plated on MM plates with diclofenac and incubated in darkness at 28 °C. Distinct colonies were reinoculated on R2A plates (R-2A agar; Fluka Analytical, Sigma-Aldrich Chemie GmbH, Merck) several times until pure cultures were obtained, and then verified for growth on MM plates with diclofenac as a sole carbon source. Finally, purified colonies were grown overnight in yeast extract broth consisting of (in g/L): 3 yeast extract and 6 peptocomplex (Biolife Italiana), then frozen in glycerol (15%) and stored at –80 °C.

### Identification of the isolated bacteria

The isolated bacteria were grown in yeast extract broth (Biolife Italiana), overnight at 28 °C and 220 rpm in darkness. The overnight cultures were then boiled for 10 min and 3 μL of boiled cells were used as a template to amplify the 16S rRNA genes using primers 27f and 1406R (*23*) and PCR conditions as described above for T-RFLP. PCR reactions were purified with QuickClean 5M PCR purification kit (GenScript USA Inc., Piscataway, NJ, USA) and sequenced by Macrogen Inc. (Seoul, Korea). Sequences of 700-800 bp were obtained. Some isolates were selected for sequencing with both forward and reverse primers (which gave approx. 300 bp of overlapping sequence) to get more reliable information about the identity of the isolates. The partial 16S rRNA gene sequences were identified by Basic Local Alignment Search Tool (BLAST; (*24*)) searches at NCBI and deposited in the GenBank database under accession numbers JF928537 to JF928555. GenBank sequences, which were identified by BLAST searches to be most similar to the isolate sequences, were downloaded and included in the phylogenetic tree reconstruction using neighbour-joining method with 500 bootstrap replicates and the Maximum Composite Likelihood evolutionary model within the MEGA v. 4 (*25*).

## RESULTS AND DISCUSSION

### Nitrification activity during the experiment

**Fig. 1:**
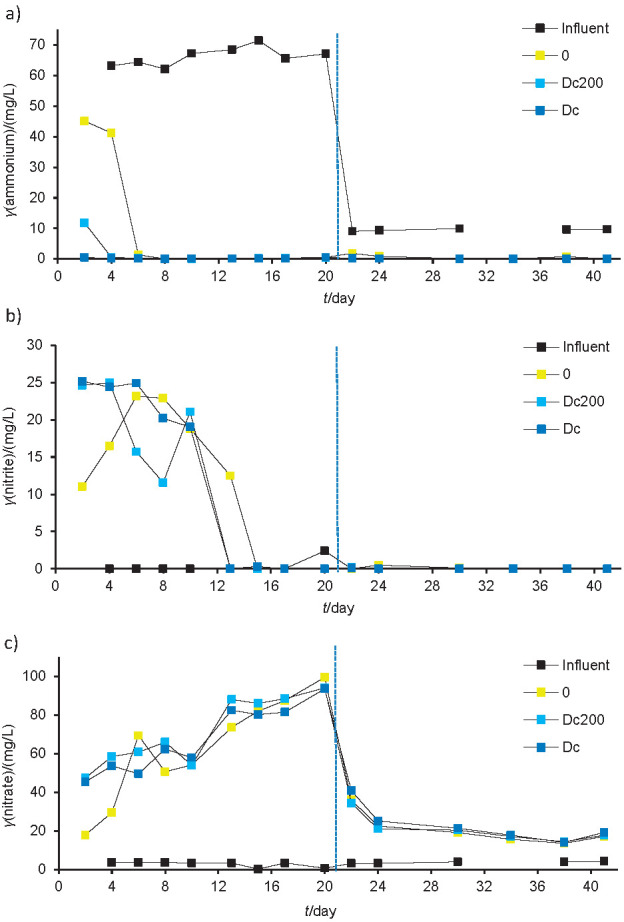
The concentrations of: a) ammonium, b) nitrite and c) nitrate in the small fed-batch reactors during the experiment. Vertical dashed lines indicate the time point where all carbon sources except diclofenac were excluded from the influent artificial wastewater. Dc=flask with increasing diclofenac concentration up to 5 mg/L, Dc200=flask with *γ*(diclofenac)= 200 μg/L, 0=flask without diclofenac, influent=artificial wastewater used to feed the reactors

Studies of xenobiotics in activated sludge generally focus on their degradation efficiency. However, it is also very important to investigate the influence of xenobiotics on microbial activities responsible for the key functions of WWTPs, such as nitrogen removal. In the previous study (*19*), a higher concentration of nitrate was detected in the reactors containing 50 μg/L of the pharmaceutical mix than in the reactors with lower concentration of pharmaceutical mix (5 or 0 μg/L), while no significant influence of pharmaceuticals on ammonium removal was observed. In this study, nitrification activity of the activated sludge in the fed-batch reactors exposed to diclofenac was monitored every two to three days after settling down the sludge by measuring ammonium, nitrite and nitrate concentrations in the medium (Fig. 1). Nitrification performance as reflected by ammonium removal was high in all batch reactors (nearly 100%), irrespective of the diclofenac concentration (Fig. 1a). A short adaptation period was observed during the first 4-6 days when the seeding sludge was transferred from the pilot reactor to the fed-batch reactors without diclofenac or its lower concentration (200 μg/L). This lag period was not observed in the fed-batch reactors with a high concentration of diclofenac (1 mg/L at the beginning). However, irrespective of diclofenac concentration, almost no ammonium (mostly less than 0.5 mg/L) remained in the fed-batch reactors after six days of incubation. A longer adaptation period for nitrite oxidation was observed as its accumulation was detected for 13-15 days (Fig. 1b). After this period, nitrite almost totally disappeared, while nitrate concentrations increased. After the first 20 days, when all carbon sources except diclofenac were excluded and the influent ammonium concentration was lower (it dropped from approx. 65 to approx. 9.5 mg/L when all carbon sources were eliminated), the concentration of nitrate fell rapidly and was relatively constant in all fed-batch reactors until the end of the experiment (Fig. 1c). In the previous study (*19*), the concentration of nitrate in the effluent of the reactors was very low (except in the reactors with 50 μg/L of pharmaceuticals). This was attributed to the denitrification in the reactors under the microaerophilic conditions that were probably present in the activated sludge flocs. In contrast, in this experiment the reactors were thoroughly shaken and flocs could not develop. However, despite different conditions in the pilot reactors and fed-batch reactors used in this experiment, ammonium removal was high in both systems and inhibition of nitrification process by diclofenac was not detected.

### Bacterial community shifts during the experiment

**Fig. 2:**
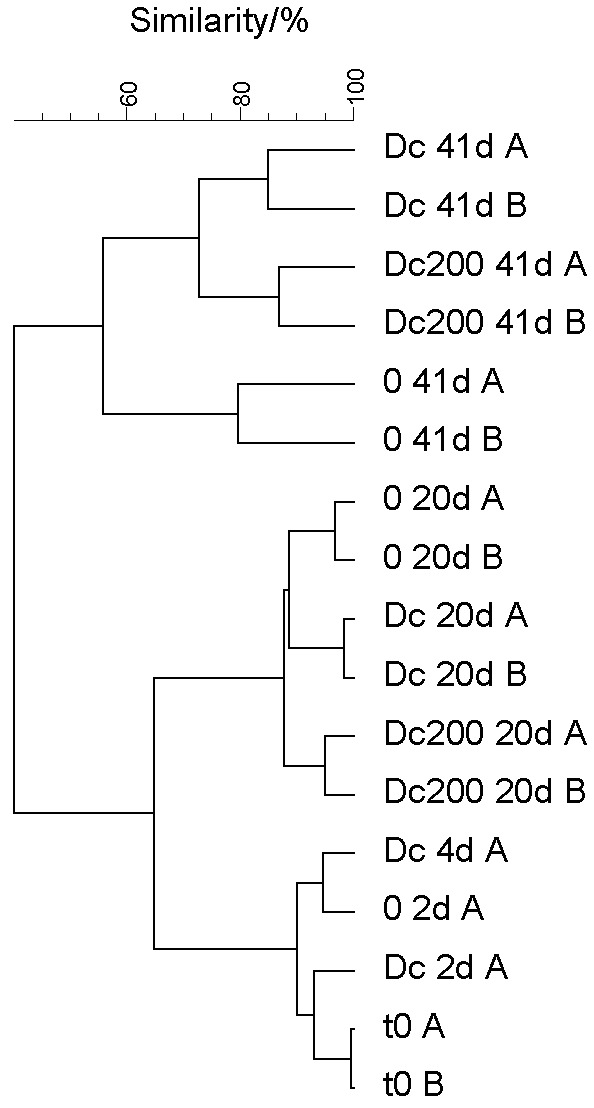
Comparison of the activated sludge bacterial community profiles treated with different concentrations of diclofenac at three different times of incubation. The dendrogram of the terminal restriction fragment length polymorphism (T-RFLP) profiles was generated based on Pearson’s correlation using the unweighted pair-group method with arithmetic means (UPGMA) method. 0=reactor without diclofenac, Dc200=reactor with *γ*(diclofenac)=200 μg/L, Dc=reactor with increasing diclofenac concentration up to 5 mg/L, t0=activated sludge used for seeding the flasks, A and B=DNA isolations from duplicate samples, 2d, 4d, 20d, 41d=samples taken after 2, 4, 20 and 41 days of the incubation

The bacterial community structure of activated sludge in the fed-batch reactors was evaluated by T-RFLP at the beginning of the experiment, after 20 days of incubation with diclofenac (0.2 or 5 mg/L) and at the end of the experiment (41 days), when the sludge was incubated for the last 21 days with diclofenac as the only carbon source. Comparison of T-RFLP profiles of bacterial communities is shown as a dendrogram in Fig. 2. Community profiles representing inoculum and the first four days of experiment (samples designated t0, 2d, 4d), clustered separately from the samples after 20 days (samples designated 20d) and after 41 days (41d) of incubation, indicating shifts in bacterial communities over time. This could partly be attributed to the adaptation to the altered conditions when transferring the sludge from the pilot reactors to the fed-batch reactors (communities diverged to approx. 65% similarity). The highest divergence (only approx. 40% similarity) was observed when all carbon sources were excluded from the artificial wastewater and diclofenac served as the only carbon source (Fig. 2). Also, the community profile of the sludge incubated without diclofenac diverged most from the other two communities at the end of the experiment. The similarity among the replicate samples was at least 80%, while similarity between fed-batch reactors with or without diclofenac at the end of the experiment was approx. 55% (Fig. 2).

**Fig. 3:**
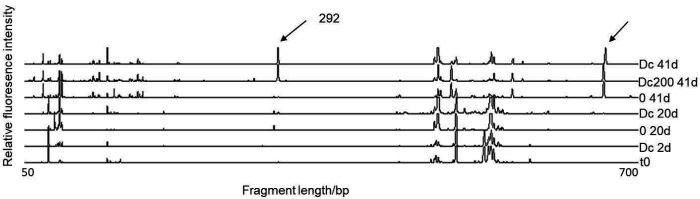
Terminal restriction fragment length polymorphism (T-RFLP) profiles of the bacterial communities in the flasks at different times of incubation. The most distinctive change in the T-RF of 292 bp (indicated by an arrow) occurred in the flasks treated with diclofenac without any other carbon source. The other T-RF indicated occurred in all flasks at the end of the experiment. t0=activated sludge used for seeding the flasks, 0=reactor without diclofenac, Dc200=reactor with *γ*(diclofenac)=200 μg/L, Dc=reactor with increasing diclofenac concentration up to 5 mg/L, 2d, 20d, 41d=samples taken after 2, 20 and 41 days of incubation experiment

Fig. 3 shows the T-RFLP profiles of some representative samples. Major differences could be observed in the profiles at the end of incubation, where at least two new peaks are evident. One of the two T-RF peaks was present in all samples at the end of the experiment (on the right end of the profiles, the length was not estimated since it was already above the range of our standard, which was 500 bp) and could be attributed to the absence of carbon sources during the last 21 days of incubation. We affiliated the most distinctive peak, which clearly appeared only in the fed-batch reactors with diclofenac and not in the control (Fig. 3), with T-RF of 292 bp. This peak was identified by *in silico* T-RFLP analyses of the clone library constructed previously (*18*), considering the T-RF drifts of *in silico* predicted and the experimental T-RF lengths of approx. 4 bp (*19*). Based on our previous analyses (*19*) the 292 bp T-RF could be affiliated with *Acidobacteria* phylum. Representatives of this phylum are very difficult to isolate (only a few representatives have been isolated so far) and are therefore not well characterized metabolically, although molecular analyses indicate their widespread presence across variety of environments (*26*, *27*).

Our results indicated that there was no significant influence of dicofenac on the bacterial community structure in activated sludge during the first 20 days of the experiment, when additional carbon was still present, even if its concentration was 5 mg/L. This is not surprising as the community has been previously adapted to a variety of pharmaceuticals. Moreover, consistently with Zhang *et al.* (*11*), who reported relatively low degradation efficiency for diclofenac in WWTPs, bacteria will preferentially degrade more easily degradable carbon source. However, a detectable change in bacterial community structure occurred when diclofenac was provided as the only carbon source and other carbon sources were removed. This change indicates that one or more microbial types became enriched in the setting and that adapted sludge has a potential for growth on diclofenac and consequently its removal. However, we cannot exclude the possibility that this shift might be the result of enrichment of certain diclofenac-tolerant community members that grow on dead biomass. Therefore, further studies are needed to characterize the species responsible for the changed pattern.

### Isolation of bacteria capable of growth on minimal agar plates with diclofenac as a sole carbon source

**Fig. 4:**
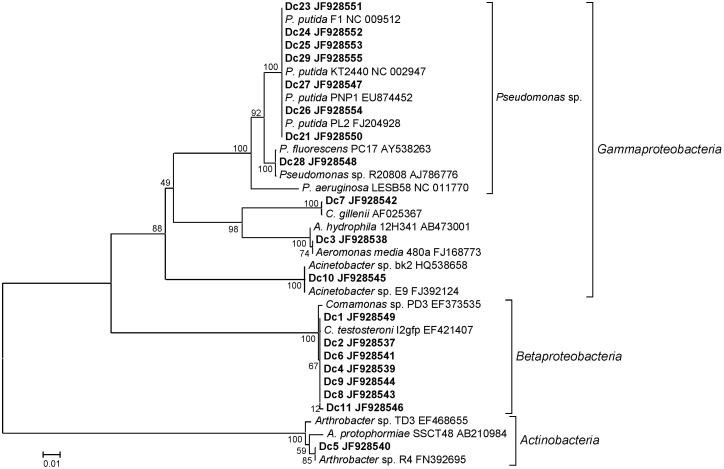
Phylogenetic tree based on partial 16S rRNA gene sequences showing the affiliations of the isolates obtained in this study (printed bold) to similar known species found by Basic Local Alignment Search Tool (BLAST) searches in the GenBank database (*22*). The percentage of replicate trees in which the associated taxa clustered together in the bootstrap test (500 replicates) is shown at the nodes

As we observed a change in microbial community structure due to diclofenac, we aimed to isolate the bacteria capable of growing on minimal medium containing diclofenac as the only carbon source. Bacteria were isolated from the original bioreactor sludge used for inoculating the fed-batch reactors and from the sludge exposed to higher concentrations of diclofenac after the 41-day experiment. From the first sample, representatives of five genera were isolated, while after incubation in the absence of other sources of carbon during the last 21 days of experiment, only two different *Pseudomonas* sp. were isolated. In Fig. 4, a phylogenetic tree indicates the affiliation of the isolates obtained in this study with the related strains found by BLAST searches of the 16S rRNA genes. Eleven isolates from the seeding sludge designated Dc1-Dc11 belonged to *Gammaproteobacteria*, *Betaproteobacteria* and *Actinobacteria*. From the activated sludge in fed-batch reactors, after enrichment experiment we isolated eight strains (Dc21-Dc28) which were taxonomically less diverse and belonged to *Pseudomonas* sp. (*Gammaproteobacteria*). Changes in the community structure before and after the enrichment with diclofenac as the only carbon source were detected by culture-independent molecular methods as well as with the selective isolation of bacteria capable of growing on diclofenac. Affiliations and identities of partial 16S rRNA genes of the isolates with the related strains are shown in Table 1. Five species isolated from the seeding sludge showed 99-100% sequence identity to related strains in the GenBank, which harbour representatives previously indicated for their potential to degrade aromatic compounds. Majority (7) of the obtained isolates were relatives of *Comamonas testosteroni*, which is able to mineralize complex and xenobiotic aromatic compounds as the sole carbon and energy source (*28*, *29*). Boon *et al.* (*30*) demonstrated that wastewater reactors augmented by *C. testosteroni* I2 *gfp* showed accelerated degradation of toxic chlorinated aromatic compound 3-chloroaniline. This strain also protected the nitrifying bacterial community, allowing for its faster recovery from the toxic shocks. *Acinetobacter* sp. and *Arthrobacter* sp. HY2 degraded *p*-nitrophenol (*31*, *32*), while *Citrobacter* strains isolated from various effluent treatment plants degraded a range of aromatic compounds and biotransformed mono- and dichlorophenols (*33*). Species from the genus *Aeromonas* are commonly reported in clinical studies, but they also degrade aromatic compounds (*34*).

**Table 1:** Identification and similarity of the isolates with the known species as found by Basic Local Alignment Search Tool (BLAST) searches in the GenBank (*22*)

Strains with sequences similar to the isolates (BLAST search)		Similarity /%	Strain information found in the GenBank
Seeding activated sludge	*Comamonas testoste*roni strain I2gfp (EF421407) *Comamonas* sp. PD3 (EF373535)	1299/1299 (100) 1297/1299 (99.9)	3-chloroaniline-degrading *Comamonas testosteroni* strain, I2gfp, isolated from wastewater phenol-degrading bacteria
	*Aeromonas media* strain 480a (FJ168773)	1309/1311 (99.9)	analysis of 16S rRNA gene mutations in a subset of *Aeromonas* strains
	*Arthrobacter* sp. R4 (FN392695) *Arthrobacter* sp. TD3 (EF468655) *A. protophormiae* SSCT48 (AB210984)	1272/1272 (100) 1276/1286 (99.2) 1274/1285 (99.1)	microcystin-degrading bacteria isolated from water 2,4,6-trinitrotoluene-degrading bacteria bacteria isolated from sewage sludge compost
	*Citrobacter gillenii* (AF025367)	1304/1305 (99.9)	*Citrobacter* phylogeny by 16S rRNA gene sequence comparison
	*Acinetobacter* sp. E-9 (FJ392124) *Acinetobacter* sp. bk_2 (HQ538658)	1261/1263 (99.8) 1299/1301 (99.9)	phenol-degrading bacteria isolated from leaf microbial communities representative strains isolated from bulking activated sludge
Sludge after enrichment	*P. putida* strain PNP-1 (EU874452) *P. putida* strain PL2 (FJ204928) *P. putida* F1 (NC009512) *P. putida* KT2440 (NC002947) *P. aeruginosa* LESB58 (NC011770)	1299/1299 (100) 1298/1299 (99.9) 1299/1299 (100) 1298/1299 (99.9) 1256/1308 (96.0)	*p*-nitrophenol-degrading bacteria isolated from soil strain capable of biodegrading polycyclic aromatic hydrocarbons isolated from polluted soil completely sequenced *Pseudomonas putida* F1, isolated from a polluted creek in Urbana, IL, USA complete genome sequence of *Pseudomonas putida* KT2440 the Liverpool epidemic strain of *Pseudomonas aeruginosa*

Although the same method of isolation was used for all isolates in this work, the isolates from the seeding sludge were more diverse than those exposed to diclofenac without other carbon sources. The seeding sludge from the reactor was exposed for 18 months to diclofenac (in a mixture of five different pharmaceuticals at concentration of 200 µg/L) with many other carbon sources always present in artificial wastewater, which enabled the growth of co-metabolizing bacteria, while in the fed-batch reactors, enrichment of bacteria able to utilize diclofenac as the sole carbon source was favoured. Seven out of eight *Pseudomonas* isolates were affiliated with *Pseudomonas putida* (Fig. 4), which is a metabolically versatile bacterium closely related to *P. aeruginosa* and important for bioremediation of sites contaminated with multiple aromatic hydrocarbons. The partial 16S rRNA gene sequence of our isolate was 100% similar to that of *P. putida* F1, which is one of the most well-studied aromatic hydrocarbon-degrading bacterial strains (*35*).

### Importance of the isolated bacteria

Many bacteria, which are able to degrade different pollutants, are being isolated from various polluted environments. Our results suggest that the same species or genera previously characterized to be metabolically versatile and able to degrade diverse aromatic compounds, emerging in the environment, might also be able to degrade diclofenac. In comparison with ibuprofen, the diclofenac phenol ring is halogenated, which contributes to the recalcitrance of this drug. However, when we performed similar enrichment experiments with ibuprofen instead of diclofenac, almost identical bacteria were isolated (as reflected from partial 16S rRNA gene sequence; data not shown). In addition, shifts in microbial community structure were very similar to the diclofenac-enriched community, as the same distinctive T-RF occurred in the presence of both pharmaceuticals when other carbon sources were absent (data not shown).

We performed T-RFLP analyses of the isolated strains to identify specific T-RFs in the total community profiles. These only represented very minor T-RFs (with low relative peak heights), suggesting that the isolated bacteria are not the most abundant in polluted environments. None of the isolates corresponded to the distinctive T-RF of 292 bp, enriched at the end of incubation with diclofenac as the only carbon source. It seems that bacteria in the activated sludge representing this T-RF cannot be isolated by the method applied here and alternative approaches will need to be devised in the future. Nevertheless, we isolated seven strains capable of growing on minimal medium with diclofenac as a sole carbon source and these represent potential models that can be applied to better understand the metabolism of diclofenac and study their potential for its removal from wastewater in monoculture and in more complex communities, such as activated sludge.

Since pharmaceuticals are usually present in wastewaters at concentrations of μg/L or less, it is possible that they do not serve as the sole carbon sources for the wastewater microorganisms and degradation in the presence of high concentrations of many different carbon sources is probably more relevant for the degradation of diclofenac in WWTPs. Gröning *et al.* (*36*) have proposed that the apparent lack of degradative potential of diclofenac as well as the failure to detect an enrichment of diclofenac-depleting microbial activity both indicate a co-metabolic nature of diclofenac transformation. Similarly, Pieper (*37*) suggested that polychlorinated biphenyl (PCB)-degrading organisms do not usually use PCBs as an energy source, but rather catabolize these substrates co-metabolically. In fact, there are a few reports in the recent years about the degradation of diclofenac: some indicate white-rot fungi as biodegraders (*38*), while the first bacterium capable of using diclofenac as its sole carbon source has been reported only recently (*39*). They showed that the isolated strain *Brevibacterium* sp. D4 could biodegrade 35% of diclofenac as a sole carbon source, while periodic feeding with acetate as a supplementary carbon source enhanced biodegradation to the levels up to 90%. Similarly, it was demonstrated that the isolated strain *Enterobacter hormaechei* D15 could biodegrade diclofenac at an elimination rate of 52.8%, while in the presence of glucose as a supplementary carbon source, degradation rate increased to approx. 82% (*40*). Our preliminary assays of diclofenac degradation by the isolated bacteria have similarly indicated better elimination rates when tests were performed in the presence of other carbon sources in addition to diclofenac. This might be a consequence of the co-metabolic nature of diclofenac degradation or merely the lower growth rate of the isolates when only diclofenac is available as a carbon source. Evidently, co-metabolism of diclofenac is more efficient than its degradation as a sole carbon source and search for bacteria in a consortium able to degrade it co-metabolically should be adapted accordingly.

## CONCLUSIONS

Our results indicate that nitrification process in small fed-batch reactors was efficient and was not detectably affected by relatively high concentrations of diclofenac (up to 5 mg/L), suggesting that the selected recalcitrant and commonly detected pharmaceutical does not influence the sensitive and important process of wastewater treatment. Moreover, bacterial communities adapted to 0.2 mg/L diclofenac did not strongly change at higher diclofenac concentrations (5 mg/L), at least under the conditions applied in our experiment, which could explain the low diclofenac degradation efficiency often observed in wastewater treatment plants. Influence of diclofenac at concentrations of 0.2 and 5 mg/L on the structure of activated sludge microbial communities was only evident when other carbon sources were not added. The isolated strains obtained in this study are very important as potential model bacteria to study bacterial diclofenac degradation. However, the disagreement between the bacterial community structure determined by the cultivation-independent approach and the bacterial isolation needs to be taken into account.
